# MRI follow-up for pancreatic intraductal papillary mucinous neoplasm: an ultrashort versus long protocol

**DOI:** 10.1007/s00261-021-03382-4

**Published:** 2021-12-18

**Authors:** Katarina Johansson, Harri Mustonen, Heini Nieminen, Caj Haglund, Tiina E. Lehtimäki, Hanna Seppänen

**Affiliations:** 1 Department of Radiology, HUS Diagnostic Center, University of Helsinki and Helsinki University Hospital, P.O. Box 340, 00029 Helsinki, Finland; 2 Department of Surgery, University of Helsinki and Helsinki University Hospital, PO Box 440, 00029 Helsinki, Finland; 3 Translational Cancer Medicine Research Program, University of Helsinki, Helsinki, Finland

**Keywords:** Pancreatic intraductal neoplasms, IPMN, MRI, Abbreviated protocol

## Abstract

**Purpose:**

To evaluate whether an ultrashort-protocol (USP) MRI including only T2-weighted HASTE axial and 3D MRCP SPACE sequences adequately measures the largest diameter of the largest cyst and the main pancreatic duct (MPD) and identifies worrisome features (WF) and high-risk stigmata (HRS) when compared to longer protocols (LP, long protocol; SP, short protocol; S-LP, short or long protocol). We also calculated reductions in costs associated with USP.

**Methods:**

This retrospective study included 183 IPMN patients. Two radiologists compared two imaging sets (USP versus S-LP) per patient, comparing the mean values of the largest cyst and MPD and agreement regarding the presence or absence of cystic or MPD mural nodules and solid pancreatic tumors. The interobserver agreement for cystic mural nodules and WF/HRS was evaluated, using the Bland-Altman plot and Cohen’s Kappa.

**Results:**

A total of 112 IPMN patients were evaluated. For detecting cysts or MPD nodules, WF/HRS, and solid pancreatic tumors, USP and S-LP coincided in 94.9%, 99.1%, 92.4%, and 99.1% of cases, respectively. Both USP and S-LP identified all true cystic mural nodules. The mean size of the largest cyst and MPD was 19.48/19.67 mm and 3.24/3.33 mm using USP versus S-LP, while the mean differences for USP versus S-LP were 0.19 mm and 0.08 mm. The USP cost was 39% of LP cost and 77% of SP. Interobserver agreement was moderate to strong.

**Conclusions:**

For IPMN surveillance, an ultrashort-protocol MRI provides nearly identical information to the more expensive longer protocols.

**Graphical abstract:**

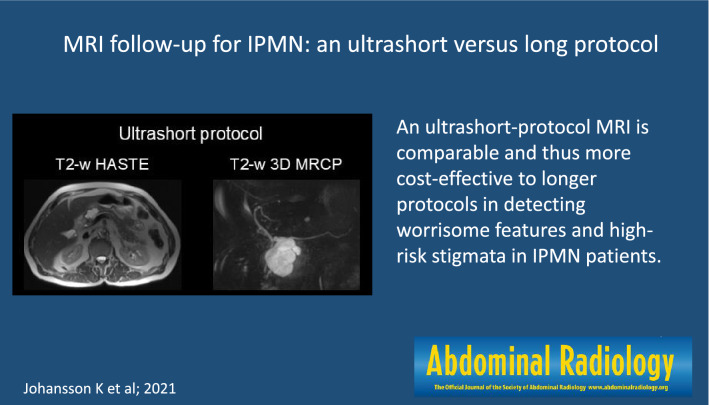

**Supplementary Information:**

The online version contains supplementary material available at 10.1007/s00261-021-03382-4.

## Introduction

The prevalence of intraductal papillary mucinous neoplasms (IPMNs) of the pancreas has increased in recent decades, partially due to better detection through the widespread use of improved resolution computed tomography (CT) and magnetic resonance imaging (MRI) [1, 2]. Reports have indicated that the prevalence of pancreatic cysts in a healthy population is 44.7–49.1% [2, 3]. IPMN changes carry a risk of malignant transformation, such that IPMN patients remain under surveillance [4]. The 2018 European guidelines recommend lifelong follow-up in patients considered fit for surgery [4]. Frequent follow-up, however, is costly and burdensome to healthcare systems.

Three types of IPMNs exist with different risk profiles for malignant transformation [5]. Branch-duct IPMNs (BD-IPMNs) carry a pancreatic malignancy incidence rate of 3.3% at 5 years following diagnosis and 15% at 15 years [6]. The highest risk of malignant transformation accompanies main-duct IPMN (MD-IPMN) and mixed-type IPMN (MX-IPMN)), with risks ranging from 38 to 68% [1, 7]. The presence of worrisome features (WF) or high-risk stigmata (HRS) is associated with an increased risk for high-grade dysplasia or cancer [4]. WF include main pancreatic duct (MPD) dilatation of 5 to 9 mm, a cyst size of ≥ 3 cm, an enhancing mural nodule of < 5 mm, thickened enhanced cyst walls, an abrupt change in the MPD caliber with distal pancreatic atrophy, and lymphadenopathy [1]. HRS includes MPD ≥ 10 mm and an enhanced mural nodule of ≥ 5 mm [1].

MRI and magnetic resonance cholangiopancreatography (MRCP) represent the primary imaging modality for IPMN surveillance given the higher contrast resolution compared to CT [4, 8] and the increased sensitivity in identifying the presence of a mural nodule or internal septations [4, 9]. MRI does not use radiation compared to CT. Follow-up using MRI aims to identify patients who present with WF or HRS.

MRI surveillance protocols vary, whereby no definitive MRI protocol is recommended [4]. The routine use of contrast material for MRI follow-up remains controversial [10]. In 2017, a short protocol (SP) without neither contrast enhancement nor diffusion sequences provided comparable information to that relying on a longer protocol (LP) [11]. At Helsinki University Hospital, IPMN patients are scanned for primary diagnosis using LP which includes contrast enhancement and diffusion imaging. Patients with WF/HRS are followed-up by LP, while those without WF/HRS undergo SP.

This study aimed to evaluate whether an even shorter MRI protocol, a so-called ultrashort protocol (USP), could provide comparable information to the longer SP and LP now routinely used at Helsinki University Hospital. Shortening the protocol would save time and reduce healthcare costs. We aimed to determine if USP is suitable for detecting cystic and MPD mural nodules and for identifying patients with WF or HRS. We also evaluated the interobserver agreement for cystic mural nodules (meaning a solid nodule arising from the wall of the cyst) and WF/HRS and the intra-observer agreement when using USP and S-LP. In addition, we calculated reductions in cost and time related to USP.

## Materials and methods

### Patient population

Our study population included 183 patients assessed for pancreatic cysts at the Helsinki University Hospital pancreatic outpatient clinic between April 2015 and December 2016.

**Fig. 1:**
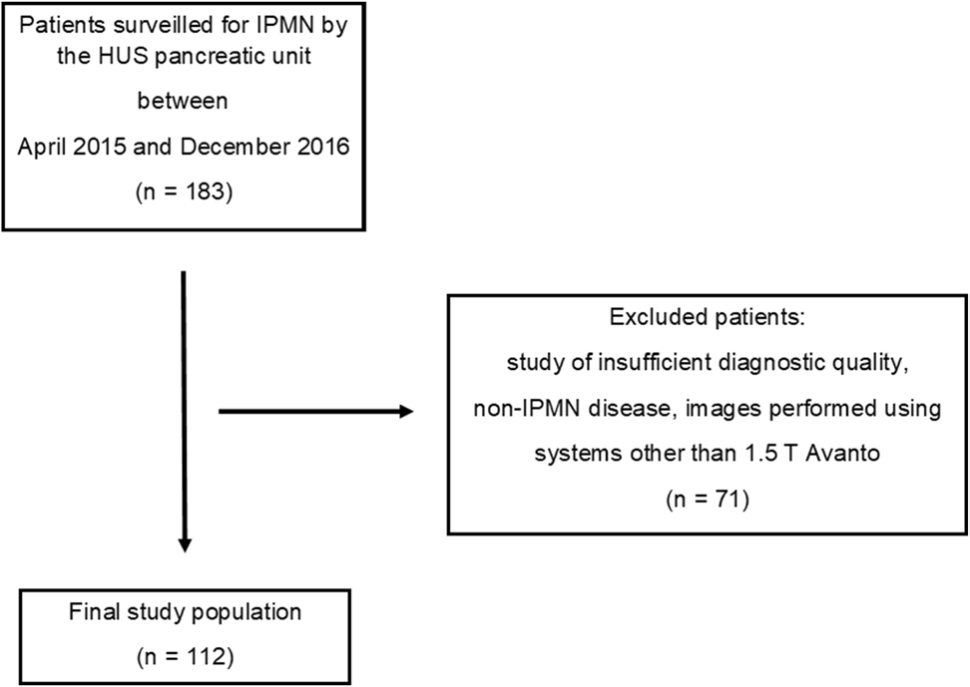
Flowchart of study participant selection

In this study, we included all patients surveilled using 1.5 Tesla (1.5 T) Magnetom Avanto MRI equipment (Siemens Healthineers). We excluded patients for whom non-diagnostic quality images were available and patients with non-IPMN disease (Fig. 1). A positive working diagnosis of IPMN was made by radiological diagnostic criteria [1].

When multiple examinations were available for one patient, we included the examination with least motion artifacts in our evaluation.

This retrospective study was registered with and approved by the Surgical Research Committee of Helsinki University Central Hospital (HUS/333/2019, extended HUS/155/2021). Because of the retrospective nature of this study and in accordance with the Act on the Secondary Use of Health and Social Data (552/2019), written informed consent of the patients was not required.

### MRI technical parameters and protocols

All MRI examinations were performed using the 1.5 T Magnetom Avanto system. Examinations performed using 3 T were excluded in order to maintain equivalency regarding image quality. Table 1 provides the technical parameters for the MRI protocols (USP, SP, and LP) and the sequences used in this study. T2-weighted half-Fourier single-shot turbo spin-echo (HASTE) axial sequences were selected for proper parenchymal visualization, and 3D MRCP sampling perfection with application-optimized contrasts using different flip angle evolution (SPACE) provided detailed MPD and cyst observation.

**Table 1:** Technical parameters of the MRI imaging protocol and sequences included in the imaging datasets for the ultrashort, short, and long protocols

Sequence, imaging plane	LP	SP	USP	Slice thickness (mm)	TE (ms)	TR (ms)	Scan time	Breathing technique
T2 HASTE axial	X	X	X	6.0	93	1400	1 min 26 s	BH
T2 HASTE fs thick slice^a^ coronal	X	X		50.0	741	4500	1 min 17 s	BH
T2 HASTE fs axial	X			6.0	93	1400	2 min 55 s	BH
T1 FLASH fs axial	X	X		5.0	2.38	178	1 min 32 s	BH
T1 2D in/opposed phase axial	X			5.0	2.38/4.76	150	49 s	BH
T2 3D SPACE MRCP^b^ coronal	X	X	X	1.0	700	2500	4 min 26 s	RT
T2 HASTE fs thin slice coronal	X	X		4.0	79	1200	1 min 50 s	BH
T1 VIBE pre-contrast axial	X			3.0	1.86	3.99	13 s	BH
T1 VIBE post-contrast^c^ axial and coronal	X			3.0 1.7	1.86 1.07	3.99 2.92	Total 5 min 13 s	BH
DWI^d^ + ADC axial	X			6.0	63	4600	3 min 41 s	FB

All sequences also included localizer and T2 TRUFI coronal sequences, for which the total time was 1 min 1 s

*ADC* apparent diffusion coefficient maps, *BH* breath-hold, *DWI* diffusion-weighted imaging, *FB* free-breathing, *fs* fat saturation, *FLASH* fast low angle shot, *HASTE* half-Fourier single-shot turbo spin-echo, *LP* long protocol, *MIP* maximum intensity projection, *MRCP* magnetic resonance cholangiopancreatography, *RT* respiratory trigger, *SP* short protocol, *SPACE* sampling perfection with application-optimized contrasts using different flip angle evolution, *TE* echo time, *TR* repetition time, *TRUFI* true fast imaging with steady-state free precession, *USP* ultrashort protocol, *VIBE* volumetric interpolated breath-hold

^a^T2 HASTE fs thick slice in six dimensions

^b^T2 3D SPACE MRCP + MIP. MIP pictures were not evaluated in USP

^c^0.1 mmol/kg body weight of gadoterate meglumine (Dotarem, Guerbet). Post-contrast: arterial phase axial, venous phase axial, venous phase coronal, and late venous phase axial. Total time: 5 min 13 s

^d^DWI *b*-values 50, 400, and 800 s/mm^2^

**Fig. 2:**
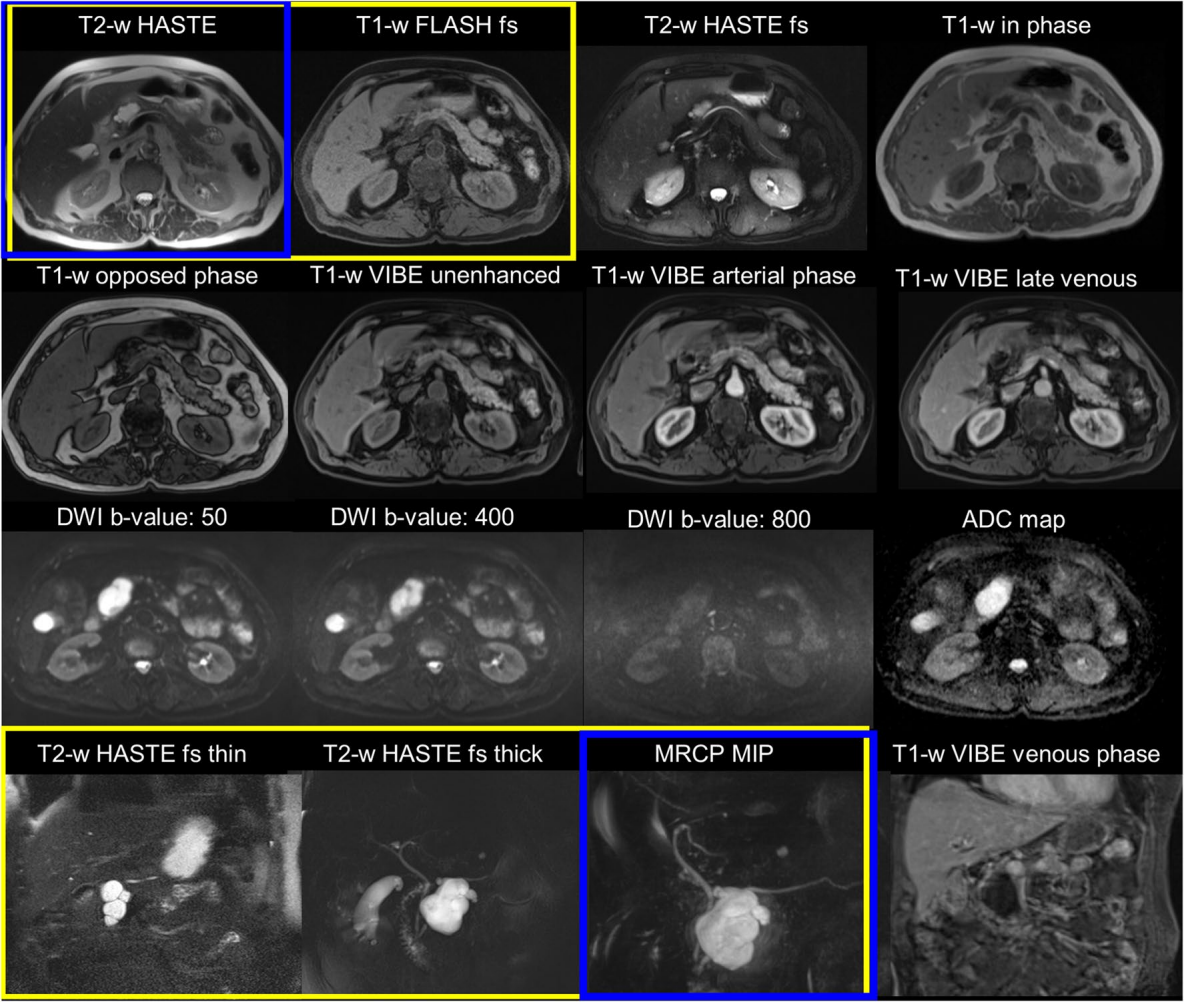
MRI imaging sets for a 79-year-old male patient under surveillance for a multicystic lesion with a mural nodule at the head of the pancreas. Blue frames represent ultrashort protocols imaging sets and yellow frames represent short protocol imaging sets. All imaging sets reflect the long protocol. MRCP MIP imaging set expresses the 3D SPACE images. *ADC* apparent diffusion coefficient maps, *DWI* diffusion-weighted imaging, *fs* fat saturation, *FLASH* fast low angle shot, *HASTE* half-Fourier single-shot turbo spin-echo, *MIP* maximum intensity projection, *MRCP* magnetic resonance cholangiopancreatography, *SPACE* sampling perfection with application-optimized contrasts using different flip angle evolution, *VIBE* volumetric interpolated breath-hold

The following MRI datasets were evaluated (Fig. 2):

- Long protocol (LP): T2-weighted HASTE axial, T2-weighted HASTE fs thin coronal, T2-weighted HASTE fs axial, T1-weighted fast low angle shot (FLASH) fs axial, T1-weighted in/opposed phase, T2-weighted 3D MRCP SPACE and maximum intensity projection (MIP), T2-weighted HASTE fs thick coronal, diffusion-weighted imaging (DWI) and apparent diffusion coefficient maps (ADC), and T1-weighted fs volumetric interpolated breath-hold (VIBE) before and after the administration of the contrast agent.
- Short protocol (SP): T2-weighted HASTE axial, T2-weighted HASTE fs thin coronal, T1-weighted FLASH fs axial, T2-weighted HASTE fs thick coronal, 3D MRCP SPACE, and MIP.
- Ultrashort protocol (USP): T2-weighted HASTE axial and 3D MRCP SPACE.

### Image analysis

Two radiologists experienced in pancreatic imaging (KJ and TL, with 5 and 12 years’ experience, respectively) independently evaluated the datasets for each patient in a random order. The radiologists, aware of the study aims, were blinded to all clinical information except for the patient’s age and gender. All USP images were analyzed first, while the S-LP images were analyzed 6 to 12 months later. Both datasets were ordered randomly so that the radiologists could not link the different protocols to a specific patient.

The following parameters were evaluated from the datasets: cyst and MPD size, cystic and MPD mural nodules, abrupt change in the MPD caliber, distal parenchymal atrophy, lymph node enlargement, and a solid pancreatic tumor (Supplementary Table 1).

In this study, the longer protocols (SP and LP) were evaluated as equivalents due to their routine use in our hospital and referral to S-LP.

### Cost and time analysis

We calculated the reduction in cost from substituting LP and SP with USP for patient surveillance through a comparison of the nominal USP cost to both the LP and SP costs based on the 2018 invoicing policy of Helsinki University Hospital. In this calculation, the following factors were considered: the use of a contrast agent, the duration of MRI suite occupancy, patient preparation, and case reading.

The sequence-based examination time for LP and SP was calculated from the MRI machine’s control panel, while MRI occupation times were collected from the imaging appointment information. The average duration of case reading times were calculated from three experienced radiologists reading IPMN controls in daily clinical workflow in 2020 in order to find out the average time used per case.

### Statistical analysis

The mural nodules were classified in a three-point scale as ‘no,’ ‘yes,’ or ‘inconclusive’. The ‘inconclusive’ was used when the radiologist was unsure if the nodule was a true nodule or sediment. In the statistical analyses, all inconclusive results regarding cystic mural nodules were coded as ‘yes’ to avoid false negatives. To detect the true cystic mural nodules, the patient reports for normal case reading were retrospectively collected by study observer (KJ), who was blinded to our study results. In cases with mural nodules also the follow-ups were read to detect which nodules actually were mucin plugs (moving mucin was seen between different scans or the mucin plug decreased in size), and which ones were more likely so-called true mural nodules. The WF and HRS analysis included cystic and MPD mural nodules, an MPD size of ≥ 5 mm, an abrupt change in the MPD caliber with distal pancreatic atrophy, a cyst of ≥ 3 cm, and/or lymphadenopathy.

The pairwise reliability between readers was measured using the Cohen’s Kappa [12]. Bootstrapping (1000 samples) was used to obtain robust bias-corrected accelerated (BCa) 95% confidence intervals (CIs) for the diameter measurements from the image analysis. The exact Clopper–Pearson 95% CIs were calculated when using proportions. In addition, we used the McNemar’s test to assess the differences in the paired contingency tables. The Bland–Altman plot was used to assess the noninferiority from the two methods using continuous measurements [13]. In order to assess the noninferiority of the Boolean data, we calculated the difference in the proportion for which a condition was detected using exact 95% CIs. The noninferiority limits for the cyst diameter were set to ± 10 mm based on previous studies [14, 15] and ± 2 mm for MDP according to the measurement accuracy. We performed all analyses using SPSS (IBM SPSS Statistics, version 25, International Business Machines Corporation) and R (version 4.0.3, Foundation for Statistical Computing).

## Results

### Study participants

From 183 patients, we excluded 53 because of the insufficient diagnostic imaging quality (primarily related to motion artifacts), 5 patients involving non-IPMN disease (pseudocysts), and 13 patients due to images captured on systems other than the 1.5 T Magnetom Avanto. The age and demographics of the excluded cases were similar to the eligible patient population (e.g., mean age ± standard deviation (SD) 68.0 years ± 11.2 in excluded cases). In total, our analysis consisted of 112 patients (70 women, 42 men, mean age ± SD 69 years ± 8). Table 2 summarizes the demographic characteristics of the study participants. Only 15 patients underwent sample taking, and one patient had surgery. The image quality was ordinary in included cases.

**Table 2:** Patient demographics

Characteristic	Total (n = 112)
Sex	
Male	42 (37.5%)
Female	70 (62.5%)
Mean age at presentation ± SD (range)	69 ± 8 (46–83)
Protocols	
Long protocol (LP) MRI	57 (51%)
Short protocol (SP) MRI	55 (49%)
Type of IPMN disease	
BD-IPMN	99 (88%)
MD-IPMN	3 (3%)
Mixed-type IPMN	10 (9%)
Surgery (distal pancreatectomy)	1
PAD low-grade dysplasia	1
EUS (cytology)	10
No atypia, benign	4
Mild atypia, benign	6
IPMN was diagnosed	4
ERP (histology)	3
No atypia, benign	3
Percutaneous fine needle aspiration or biopsy	2
No atypia, benign	1
Insufficient sample	1
Mortality	2
Pancreatic cancer	1
Other causes	1

*BD-IPMN* branch-duct intraductal papillary mucinous neoplasia, *ERP* endoscopic retrograde pancreatography, *EUS* endoscopic ultrasound, *IPMN* intraductal papillary mucinous neoplasia, *MD-IPMN* main-duct intraductal papillary mucinous neoplasia, *SD* standard deviation

### Image analysis

Table 3 presents the results for the parameters on WF/HRS, with special attention placed on cystic mural nodules and the MPD mural nodules. In addition, we evaluated solid pancreatic tumors (see Table 3) because of the possible progression of IPMN and recognizing the additional risk of a separate malignancy elsewhere in the pancreas. Overall, for cystic mural nodules, MPD mural nodules, and solid pancreatic tumor, we found agreement between the longer protocols (S-LP) and the USP in 94.9%, 99.1%, and 99.1%, respectively, of all cases. For WF/HRS, the overall agreement between S-LP and USP reached 92.4%. Table 3 also provides the difference in the proportion of detected cases between S-LP and USP, for which we used 95% CIs to assess the noninferiority between methods. We identified no significant systematic bias in the difference between methods and detected no difference between the confidence intervals. When the noninferiority limits exceed the confidence interval the result can be considered noninferior. However, we did not define these limits, because there are no published clear limits from previous studies, although the magnitude of the interrater error falls within a similar limit.

**Table 3:** Agreement between the longer and ultrashort protocols for the absence or presence and detected cases of parameters cystic mural nodules, main pancreatic duct mural nodules, solid pancreatic tumor, and worrisome features or high-risk stigmata for readers 1, 2, and overall and interobserver

Parameter	Overall	Interobserver	Reader 1	Reader 2
MN^c^	94.9		96.4 (106/110) [92.2, 99.4]	93.5 (101/108) [92.1, 99.4]
S-LP detected cases			13.6	17.6
USP detected cases			15.5	16.7
Difference^a^			− 1.8 [− 6.9, 2.6]	0.9 [− 4.7, 6.8]
ΔS-LP^b^		3.7 [− 1.0, 9.6]		
ΔUSP^c^		0.9 [− 4.7, 6.8]		
MN^MPD^	99.1		100 (112/112) [96.8, 100.0]	98.2 (110/112) [93.7, 99.8]
S-LP detected cases			0.9	1.8
USP detected cases			0.9	1.8
Difference^a^			0.0 [− 3.3, 3.3]	0.0 [− 4.1, 4.1]
ΔS-LP^b^		0.9 [− 3.3, 5.5]		
ΔUSP^c^		0.9 [− 3.3, 5.5]		
Solid Tumor	99.1		99.1 (111/112) [95.1, 100.0]	99.1 (111/112) [95.1, 100.0]
S-LP detected cases			1.8	2.7
USP detected cases			0.9	1.8
Difference^a^			0.9 [− 2.5, 4.9]	0.9 [− 2.5, 4.9]
ΔS-LP^b^		0.9 [− 2.5, 4.9]		
ΔUSP^c^		0.9 [− 2.5, 4.9]		
WF/HRS	92.4		96.4 (108/112) [91.1, 99.0]	88.4 (99/112) [81.0, 93.7]
S-LP detected cases			31.3	36.6
USP detected cases			33.0	33.9
Difference^a^			− 1.8 [− 6.8, 2.5]	2.7 [− 4.1, 9.8]
ΔS-LP^b^		5.4 [− 0.8, 12.4]		
ΔUSP^c^		0.9 [− 5.1, 7.0]		

Unless otherwise specified, data are percentages. Data in parentheses are absolute numbers detected, and data in brackets are 95% confidence intervals

*HRS* high-risk stigmata, *MN*^*c*^ cystic mural nodules, *MN*^*MPD*^ main pancreatic duct mural nodules, *S-LP* longer protocols (short or long protocol), *USP* ultrashort protocol, *WF* worrisome features

^a^Difference refers to the difference in the detected cases between methods

^b^Indicates the interobserver difference between detected cases between longer protocols

^c^Indicates the interobserver difference between detected cases between ultrashort protocols

Supplementary Table 2 summarizes how accurately the cystic mural nodules were identified using USP and S-LP. All true cases were identified using USP and S-LP. Reader 1 suspected that three more patients had a mural nodule using USP and one more using S-LP. Reader 2 suspected that four more patients had a mural nodule using USP and five using S-LP. Since the sensitivity reached 100% for both methods, these could not be compared. However, we could calculate the difference in specificity using the CIs, demonstrating how accurately the difference could be defined from these data.

**Fig. 3:**
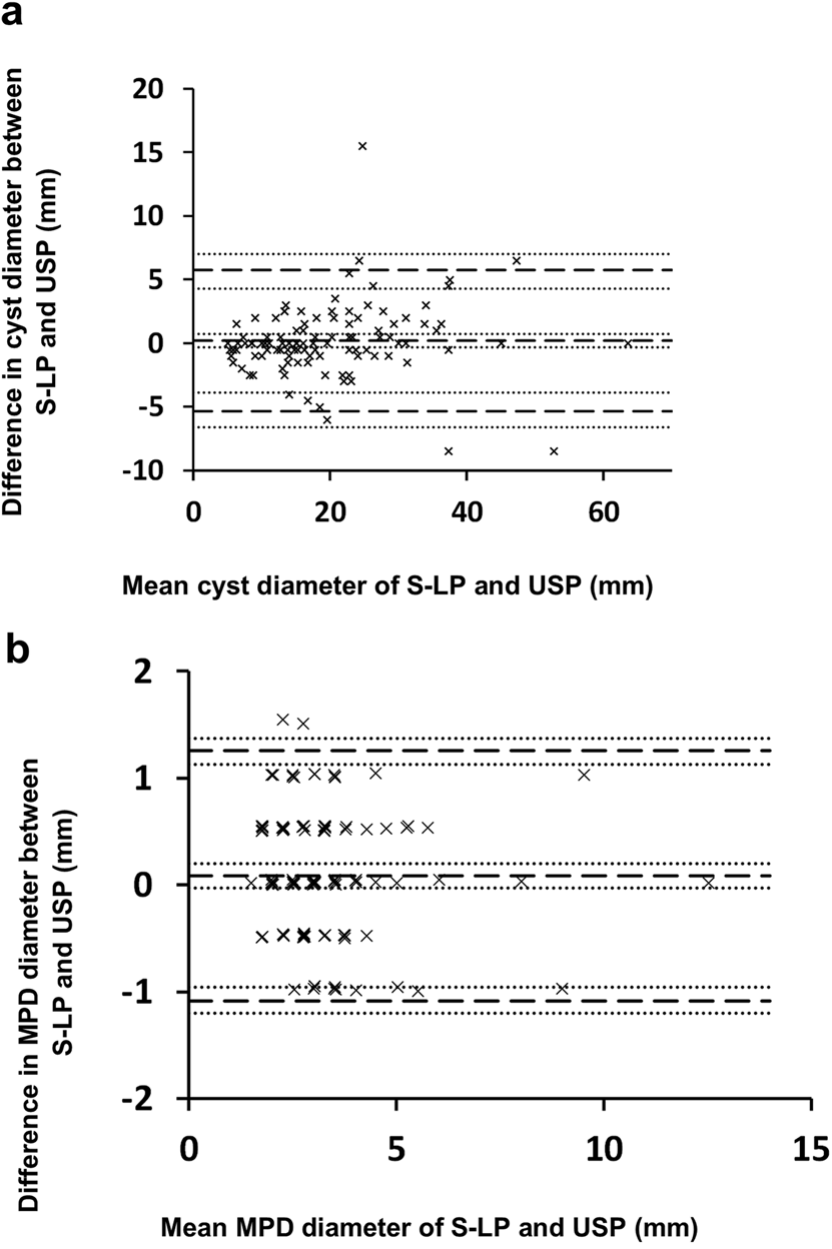
Bland–Altman plots comparing mean cyst diameter (**a**) and main pancreatic duct diameter (MPD) (**b)** from the longer protocols and ultrashort protocol. We found no significant difference when comparing the means between methods or in the visible trend in cyst diameter (**a**) or MPD diameter (**b**). The limits of agreement for the difference ranged from − 5.4 mm (− 6.6, 95% lower confidence limit, LCL) to 5.7 mm (7.0, 95% upper confidence limit UCL) for the largest cyst and from -1.1 mm (− 1.2, 95% LCL) to 1.3 mm (1.4, 95% UCL) for MPD. These limits lie within the noninferiority limits of ± 10 mm for the largest cyst and ± 2 mm for MPD. The lower and dashed lines from above indicate the upper limit of agreement, the mean value of agreement, and the lower limit of agreement. The dotted lines represent the 95% bootstrapped confidence intervals for the agreement limits and the mean difference between the methods, indicating the accuracy of these estimates. The limits of agreement are set at ± 1.96 standard deviations (SDs). *MPD*, main pancreatic duct; *S-LP*, longer protocols (short or long protocol); *USP*, ultrashort protocol

The results for the mean value of the largest cyst and main pancreatic duct appear in Table 4. The largest cyst was 19.67 mm in size according to S-LP and 19.48 mm based on USP. This 0.19-mm difference was not statistically significant, and the CIs show how accurately the difference can be determined from these data. The mean MPD value was 3.33 mm using S-LP and 3.24 mm using USP, a difference (0.08 mm) which was not statistically significant. The Bland–Altman plots provide a comparison of the USP and S-LP methods (Fig. 3). These plots reveal no significant bias in the difference between methods nor did we detect a visual trend as the size increased. The limits of agreement for the difference using CIs ranged from − 6.6 to 7.0 mm and from -1.2 to 1.4 mm for the largest cyst and MPD, respectively. Over 97% of all observations fell within these limits. Furthermore, these limits fell within the defined noninferiority limits of ± 10 mm and ± 2 mm for the cyst and MPD, respectively.

**Table 4:** Comparison between the longer protocols (S-LP) and the ultrashort protocol (USP) for the parameters mean cyst diameter and main pancreatic duct diameter (MPD) for readers 1 and 2 and overall

Reader	Parameter	S-LP	USP	Δ
		Mean (95% CI)	Mean (95% CI)	Mean (95% CI)
Overall	Cyst, mm	19.67 (17.87–21.76)	19.48 (17.70–21.67)	0.19 (− 0.31–0.70)
	MPD	3.33 (3.07–3.61)	3.24 (2.98–3.53)	0.08 (− 0.03–0.20)
Reader 1	cyst, mm	19.86 (18.02–22.09)	19.66 (17.75–21.84)	0.20 (− 0.55–0.92)
	MPD	3.27 (2.96–3.59)	3.33 (3.04–3.65)	− 0.06 (− 0.21–0.09)
Reader 2	Cyst, mm	19.48 (17.73–21.47)	19.29 (17.46–21.43)	0.18 (− 0.42–0.76)
	MPD	3.38 (3.13–3.68)	3.15 (2.88–3.42)	0.23 (0.05–0.41)

For overall within the subject difference with standard deviation and IQR are: mean 0.19 mm, SD 0.27 mm, median 0, IQR − 1–1.5 mm for cyst size, and mean 0.08 mm, SD 0.06 mm, median 0, IQR − 0.5–0.5 mm for MPD

We used bootstrapping to calculate the 95% confidence intervals (CIs). *CI* confidence interval, *IQR* interquartile range, *MPD* main pancreatic duct diameter, *SD* standard deviation, *S-LP* longer protocols (short or long protocol), *USP* ultrashort protocol, *Δ* difference between mean values for S-LP and USP

The intra-observer agreement between USP and S-LP was strong for the cystic mural nodules and almost perfect for WF/HRS for reader 1 and moderate for reader 2 (Table 5). The interobserver agreement using USP was strong for WF/HRS comparing readers and moderate for the cystic mural nodules. The interobserver agreement using S-LP was moderate for both WF/HRS and the cystic mural nodules.

**Table 5:** Intra- and interobserver agreement between readers regarding cystic mural nodule, worrisome features, or high-risk stigmata

Parameter	Cohen’s Kappa			
	Reader 1	Reader 2		
	Intra-USP vs S-LP	Intra-USP vs S-LP	Inter-USP	Inter-S-LP
MN^c^ (95% CI)	0.85 (0.71, 0.99)	0.77 (0.61, 0.93)	0.76 (0.59, 0.93)	0.79 (0.63, 0.95)
WF/HRS (95% CI)	0.92 (0.84, 0.99)	0.75 (0.62, 0.88)	0.82 (0.71, 0.93)	0.76 (0.64, 0.89)

WF/HRS included cystic and MPD mural nodules, an MPD size of ≥ 5 mm, an abrupt change in the MPD caliber with distal pancreatic atrophy, a cyst of ≥ 3 cm, and/or lymphadenopathy

For the Cohen’s Kappa, values > 0.90 indicate almost perfect agreement, values of 0.80–0.90 indicate strong agreement, and values of 0.60–0.79 indicate a moderate agreement

*CI* confidence interval, *HRS* high-risk stigmata, *MN*^*c*^ cystic mural nodule, *MPD* main pancreatic duct, *S-LP* longer protocols (short or long protocol), *USP* ultrashort protocol, *WF* worrisome features

Supplementary Table 3 summarizes how similar the WF/HRS observation was intra-observationally. Reader 1 suspected three more WF/HRS cases using USP compared with S-LP. Using USP, reader 1 found one patient with an abrupt change in the MPD caliber with distal pancreatic atrophy and an MPD diameter of 6 mm not observed in the LP analyses. The second patient exhibited an MPD diameter between 4 and 5 mm and a third patient was suspected of having an inconclusive cystic mural nodule. Using S-LP, reader 1 found one more case than through USP: the largest cyst was measured differently (31 vs. 8 mm). Reader 2 suspected five more WF/HRS cases using USP than when using S-LP. Two patients exhibited an MPD diameter of between 4 and 5 mm, two patients were suspected of having a cystic mural nodule (one was definitive and the other was inconclusive), and the largest cyst in one patient was measured differently (29 vs. 30 mm, S-LP vs. USP, respectively). Reader 2 suspected eight more WF/HRS cases using S-LP than when using USP. In one patient, the largest cyst was measured differently (26 vs. 34 mm, USP vs. S-LP, respectively), four patients were suspected to have an inconclusive cystic mural nodule, and three patients featured an MPD diameter measured as 4 to 5 mm, 4 to 6 mm, and 2 to 5 mm, respectively.

### Cost and time analysis

The cost of USP was estimated as 77% that of SP and 39% that of LP. The contrast-enhanced LP cost was €514 and the SP cost was €261, while the approximate USP cost was €201.

The total sequence-based examination time was 23 min for LP, 13 min for SP, and 7 min for USP. At Helsinki University Central Hospital, an LP occupies an MRI suite for 45 min, while SP requires 20 min. We calculated that USP would occupy an MRI suite for 15 min and, therefore, in 3 h, four patients could be scanned using LP, nine using SP, and 12 using USP.

The case reading took on average 15 min 30 s using LP, 12 min 30 s using SP, and approximately 10 min using USP.

## Discussion

One major finding from this study is that USP is comparable to longer protocols for the radiological assessment in patients under surveillance for IPMN. When reviewing interobserver agreement of cystic mural nodules, MPD mural nodules, and solid pancreatic tumors, readers reached the same conclusions using USP compared to S-LP in 94.9%, 99.1%, and 99.1%, respectively, of cases. Our study yielded similar results from a previous study by Pozzi-Mucelli et al. [10], where agreement for cystic and MPD mural nodules was 93% and 98%, respectively. Among findings for genuine cystic mural nodules, USP performed as well as S-LP. All matching cystic mural nodules were identified in each case read. Reader 1 suspected a mural nodule in two more cases when using USP than when using S-LP, although reader 2 suspected one less case when using USP than when using S-LP. We also found that the intra-observer agreement for cystic mural nodules was strong for reader 1 and moderate for reader 2, and that the interobserver agreement was moderate for both USP and S-LP. These results indicate that USP yields nearly identical results compared to S-LP and can be used in clinical practice.

When examining WF or HRS, the readers reached similar conclusions when evaluating USP and S-LP in 92.4% of cases. The intra-observer agreement was nearly perfect for reader 1 and was moderate for reader 2, while the interobserver agreement was strong for USP and moderate for S-LP. Reader 1 identified one case with abrupt change in the MPD caliber with distal parenchymal atrophy only using USP, not with S-LP. But in one case reader 1 analyzed different cysts as the largest one by using USP and S-LP leading to different measurements (8 mm and 31 mm). Reader 2’s measurements exhibited mostly clinically insignificant variations in cyst size and MPD measurements. Yet, in one patient, the MPD diameter was measured differently (2–5 mm) and an abrupt change in the MPD caliber was not identified using USP. Reader 1 also did not identify this finding using S-LP, indicating that this specific case was particularly difficult to evaluate clinically. For this specific patient, a follow-up examination at 2 years and 7 months after the initial imaging was analyzed, which indicated stable disease. Because reader 2 identified an insignificant variation between MPD and cyst measurements in a total of five cases and most of the additional cystic mural nodule observations were inconclusive when using USP and S-LP, we consider these findings insignificant. The average difference, then, in the percentage of WF/HRS detected between methods fell below 3% for both readers. In addition, the intra- and interobserver agreement results were comparable regardless of the protocol used and fell within a normal variation for this kind of multivariate image analysis. These results indicate that USP and S-LP are comparable for detecting WF or HRS in IPMN patients.

For the size-related parameters, specifically the mean cyst diameter and MPD, the overall difference between the two protocols was 0.19 mm and 0.08 mm, respectively. These data lie within the defined noninferiority limits. Thus, both S-LP and USP appear to provide noninferior results appropriate for clinical use. Therefore, dimensional changes in both lesion and MPD during surveillance can be reliably detected using USP. The noninferiority limits for a cyst were set to ± 10 mm [14, 15]. Dunn et al. demonstrated that a significant and frequent interobserver variability exists in measuring pancreatic cystic lesions [14]. In our study, the SD for cyst size was only 0.27 mm while it was 3.27 mm in the study by Dunn et al. Also, no difference between measurements exceeded 50% in our study when in their study some measurements exceeded 50% [14]. In the study by Maimone et al., the interquartile range (IQR) for cyst size was 3.5 mm while it was only 2.5 mm in our study [15]. Furthermore, the noninferiority limits for MPD were set to ± 2 mm given that we documented an MPD width within a 1-mm accuracy, leading to rounding from 1.4 to 1 mm and from 2.5 to 3 mm, indicating that a 1.1-mm difference led to a 2-mm difference.

USP should not replace LP for the primary characterization of IPMNs. USP could be used for the surveillance of BD-IPMN patients who have no WF or HRS characteristics and have been carefully evaluated. If WF or HRS characteristics develop during surveillance, performing an additional LP becomes necessary. This study warrants further research to evaluate if USP can also detect solid tumors based on the knowledge that IPMN patients carry a higher risk for cancer elsewhere in the pancreas [16]. We argue that the primary diagnosis should rely on LP. Then, if a patient exhibits no signs of WF or HRS, follow-up could rely on USP.

To calculate the cost and time reduction when substituting S-LP with USP, we used two approaches to demonstrate the magnitude of the effect. Given that the estimated USP cost is about 77% that of SP and 39% that of LP, the total cost reduction associated with using USP seems obvious. In addition, S-LP is more time-consuming than USP. We calculated that USP would occupy an MRI suite for 15 min. The reductions in cost and time resulting from substituting S-LP with USP would allow for a greater number of patient scans. In addition, case reading is faster with fewer sequences.

In previous study by Pozzi-Mucelli et al., one limitation resulted from the different slice thicknesses from differences in MRI protocols used in their study [10]. We avoided this problem because we used the same MRI machine. Our study was standardized by using examinations performed with 1.5 T. The measured qualities are likely equal both in 1.5 T and in 3.0 T machines, but further research is needed. However, our study carries some limitations. First, the primary aim of our study was to determine if readers could report similar information with USP and S-LP in the same patient, rather than analyzing the sensitivity or specificity of these protocols. Second, we conducted a retrospective study that included patients in an IPMN surveillance program for whom a histopathological confirmation was unavailable [17]. Approximately 30% of our patients had WF/HRS but only 14% of the population underwent sample taking. Our patient population consisted of elderly patients which may explain that they were frequently treated conservatively. Third, almost 29% of patients were excluded because of the insufficient diagnostic imaging quality. Especially in elderly patients, the breathing and movement artifacts are an everyday problem. In everyday clinical practice, the evaluation of the image quality is an important step of the image interpretation, and in case of non-diagnostic image quality, renewal of the examination or other imaging technique (e.g., CT) should be considered. We hope that the shorter USP examination time (sequence-based time is only 7 min) will reduce this problem. Finally, the calculated cost reduction from substituting S-LP with USP relied on the cost difference between these three protocols estimated at Helsinki University Hospital, and, therefore, variations in prices may exist between hospitals and countries.

In conclusion, an ultrashort MRI protocol is suitable for IPMN surveillance, which could relieve healthcare system burdens without losing any essential information. An ultrashort protocol should be considered as an alternative for IPMN surveillance when a patient does not have WF or HRS. The goal is to identify patients who develop WF or HRS during surveillance. While possible false-positive findings may occur, it is more important to have a low threshold for suspected WF/HRS than to not suspect them at all.

## Supplementary Information

Below is the link to the electronic supplementary material.

Supplementary file1 (DOCX 16 kb)

Supplementary file2 (DOCX 15 kb)

Supplementary file3 (DOCX 13 kb)

## Data Availability

Not applicable.
