# Supplementary material for: MRI follow-up for pancreatic intraductal papillary mucinous neoplasm: an ultrashort versus long protocol
Source: Abdom Radiol (NY). 2021 Dec 18;47(2):727–37. doi: 10.1007/s00261-021-03382-4 (PMC8807431; doi:10.1007/s00261-021-03382-4)
Supplement: Supplementary file 1 — Supplementary file1 (DOCX 16 kb) [file 261_2021_3382_MOESM1_ESM.docx]

**Supplementary Table 1** Parameters evaluated

| Parameter |  |  |
| --- | --- | --- |
| Cysts |  |  |
|  | amount | no cysts, solitary, multifocal |
|  | localization | uncinate process, head, neck, body, tail |
| Largest cyst or multicystic lesion |  |  |
|  | maximum diameter | mm |
|  | appearance | one cyst, multiple |
|  | localization | uncinate process, head, neck, body, tail |
|  | mural nodule | no, yes, inconclusive |
|  | size of the mural nodule | mm |
|  | thickening of the cyst wall | no, yes, inconclusive |
|  | communication to MPD | no, yes, inconclusive |
|  | internal septation | no, yes, inconclusive |
| WF in the other cysts |  | no, yes |
|  | mural nodule size | mm |
| MPD |  |  |
|  | dilatation | no, yes |
|  | cause of the dilatation | MX-IPMN, MD-IPMN, inconclusive |
|  | width at the broadest point | mm |
|  | location of the broadest point | uncinate process, head, neck, body, tail |
|  | change of caliber | no, yes |
|  | abrupt change to the caliber | no, yes |
|  | distal parenchymal atrophy | no, yes |
|  | mural nodule in the MPD | no, yes, inconclusive |
| Type of IPMN disease |  | MD-IPMN, BD-IPMN, MX-IPMN, unspecified |
| Pancreatic parenchyma normal |  | no, yes |
|  | why not normal | by text |
| Solid tumor in the pancreas |  | no, yes, inconclusive |
| Dilatation of the bile duct |  | no, yes |
|  | diameter | mm |
| Lymph node enlargement |  | no, yes |
| Invasion |  | no, metastasis, blood vessel invasion, growth beyond the pancreas |

*BD-IPMN*, branch-duct IPMN; *IPMN*, intraductal papillary neoplasia of the pancreas; *MD-IPMN*, main-duct IPMN; *MPD*, main pancreatic duct; *MX-IPMN*, mixed-type IPMN; *WF*, worrisome features.
