# Supplementary material for: MRI follow-up for pancreatic intraductal papillary mucinous neoplasm: an ultrashort versus long protocol
Source: Abdom Radiol (NY). 2021 Dec 18;47(2):727–37. doi: 10.1007/s00261-021-03382-4 (PMC8807431; doi:10.1007/s00261-021-03382-4)
Supplement: Supplementary file 2 — Supplementary file2 (DOCX 15 kb) [file 261_2021_3382_MOESM2_ESM.docx]

**Supplementary Table 2** Cystic mural nodules. Comparing longer protocols and the ultrashort protocol for the visualization of nodules by readers 1 and 2 and normal case reading

| Parameter | Normal case reading | Reader 1 |  | Reader 2 |  |
| --- | --- | --- | --- | --- | --- |
|  |  | **S-LP** | **USP** | **S-LP** | **USP** |
| All nodules (Absolute numbers) | 14 | 15 | 17 | 19 | 18 |
| Matching nodules | 14 | 14 | 14 | 14 | 14 |
| Sensitivity % |  | 100 | 100 | 100 | 100 |
| Specificity % (95% CI) |  | 98.9 (96.9, 100) | 96.8 (93.3, 100) | 94.7 (90.1, 99.2) | 95.7 (91.7, 99.3) |
| Δspecificity* (95% CI) |  | -2.1 (-3.2, 7.5) |  | 1.1 (-6.8–9.0) |  |
| *P* value |  | 0.32 |  | 0.71 |  |

All matching nodules were found using USP and S-LP. *CI*, confidence intervals; *S-LP*, longer protocols (short or long protocol); *USP*, ultrashort protocol.

*Δspecificity is the difference in specificity between S-LP and USP methods. The difference between methods was calculated using the McNemar’s test.
