# Supplementary material for: MRI follow-up for pancreatic intraductal papillary mucinous neoplasm: an ultrashort versus long protocol
Source: Abdom Radiol (NY). 2021 Dec 18;47(2):727–37. doi: 10.1007/s00261-021-03382-4 (PMC8807431; doi:10.1007/s00261-021-03382-4)
Supplement: Supplementary file 3 — Supplementary file3 (DOCX 13 kb) [file 261_2021_3382_MOESM3_ESM.docx]

**Supplementary Table 3** Intra-observer agreement or disagreement for worrisome features or high-risk stigmata

|  |  | Reader 1 S-LP |  |
| --- | --- | --- | --- |
|  |  | no | yes |
| Reader 1 USP | no | 74 | 1 |
|  | yes | 3 | 34 |

|  |  | Reader 2 S-LP |  |
| --- | --- | --- | --- |
|  |  | no | yes |
| Reader 2 USP | no | 66 | 8 |
|  | yes | 5 | 33 |

Data are numbers. *S-LP*, longer protocols (short or long protocol); *USP*, ultrashort protocol.
